# Development and Validation of an Autonomy Questionnaire for Chinese Adolescents From the Perspective of Network Culture

**DOI:** 10.3389/fpsyg.2022.810140

**Published:** 2022-02-28

**Authors:** Yi Li, Hong Chen, Yue-li Zheng, Ling-ling Wu, Cui-ying Fan

**Affiliations:** Key Laboratory of Adolescent Cyberpsychology and Behavior, Ministry of Education, School of Psychology, Central China Normal University, Wuhan, China

**Keywords:** adolescents, autonomy, contemporary China, questionnaire development, network culture

## Abstract

This study developed a measure of autonomy for adolescents in contemporary China. First, data from 44 interviewees—40 secondary school students, 2 parents, and 2 secondary school teachers—were used to explore the connotation and theoretical structure of autonomy in adolescents in China. Next, a preliminary Adolescent Autonomy Questionnaire was created from the interview data and administered to 775 secondary school students. Exploratory factor analysis and confirmatory factor analysis (CFA) were conducted to verify the factor structure. Finally, 614 secondary school students completed the Adolescent Autonomy Questionnaire, Personal Growth Initiative Scale-II, and Adolescence Ego Identity Crisis Scale to evaluate criterion validity. The final version of the Adolescent Autonomy Questionnaire included 16 items and four subscales: autonomous decision-making, autonomous regulation, autonomous protection, and autonomous problem-solving. The total variance of the cumulative interpretation questionnaire was 62.54%. The CFA results showed that the four-factor model fits the data well: *χ*^2^/df = 2.340, CFI = 0.949, RMSEA = 0.042, SRMR = 0.046. Evaluation of the psychometric properties of the Adolescent Autonomy Questionnaire provided support for the reliability and validity of the measure. Thus, it serves as an effective measurement tool for assessing the autonomy of adolescents in China.

## Introduction

Autonomy is a fundamental attribute of human beings as actors, which is rooted in daily life activities (Lin, 2017). In the Outline of China’s National Plan for Medium and Long-Term Education Reform and Development (2010–2020), autonomous learning (Earl et al., 2017) and autonomous development are noted as important components of students’ personality and social development (Charry et al., 2020). Etymologically, “autonomy” is a complex word; it is a combination of the Greek words *auto* (self) and *nomos* (law), meaning to live by one’s own rules. Human autonomy deals with the elements of freedom, choice, independence, desire, emotion, reason, and self-control (Steinberg and Silverberg, 1986). Previous studies on autonomy have discussed its psychological structure from the perspectives of classical psychoanalytic, self-determination, social learning, and authority control theories, and the cognitive model of depression (Campbell et al., 2003; Ryan and Deci, 2006). For example, Hill and Holmbeck (1986) divided autonomy into psychological and interpersonal dimensions, which are expressed as behavioral autonomy, emotional autonomy, and cognitive autonomy. Anderson et al. (1994) developed the Worthington Autonomy Scale and argued that autonomy includes four aspects: family loyalty autonomy, emotional autonomy, behavioral autonomy, and value autonomy. Noom et al. (2001) categorized adolescent autonomy into attitudinal, emotional, and functional autonomy. Beyers et al. (2003) categorized adolescent autonomy into four dimensions: connectedness, separation, detachment, and agency. Bekker and Van Assen (2006) developed a short form of the Autonomy-Connectedness Scale, which divides autonomy into sensitivity to others, capacity for managing new situations, and self-awareness. The Self-Determination Theory (SDT, Ryan and Deci, 1985), which adopts a macro perspective, based on a positive psychology orientation, explores the reasons behind individual behavior. SDT proposes that autonomy is a determinant of individual behavior and that autonomous behavior embodies the highest level of motivational internalization and plays a positive role in promoting individual adaptation (Deci et al., 2017). The theory also addresses individuals’ development of autonomy and the impact of specific biological and environmental conditions (Ryan and Deci, 2000). Compared to other theories, SDT has a more integrated understanding of autonomy. Empirical studies based on SDT have validated the benefits of autonomy on individual behavior, relationships, and social adjustment in different settings, such as home, school, workplace, and healthcare, where autonomy is seen as a positive potential for enhancing individual energy (Earl et al., 2017; Cook et al., 2018; Xiang and Liu, 2018).

The realization of autonomy is a universal, cross-cultural psychological need, and the development of individual autonomy is intricately linked to one’s cultural values (Keller, 2012). To study autonomy as a developmental indicator within the contemporary collectivist culture of China, we cannot simply borrow or revise research tools from other countries. We need to consider how an individual’s independence adapts to and coexists with the social environment. Xia and Huang (2012) distinguished the self-supporting personality, which is more consistent with Chinese cultural attributes, from Western autonomy. They proposed that the self-supporting personality refers to comprehensive personality characteristics that help individuals solve the problems of basic survival and development they encounter on their own, and includes the personal and interpersonal traits of independence, initiative, responsibility, flexibility, and openness. According to Lin’s (2017) study on the core competencies of student development, students’ autonomous development places a heavy emphasis on their ability to effectively manage their own learning and life, which includes six essential points: pleasure in learning, diligent reflection, information awareness, valuing life, healthy personality, and self-management.

Adolescence has been described as an important period during which individuals can explore and examine their own characteristics and achieving a sense of autonomy is considered a key developmental task during this period (Oudekerk et al., 2015). Autonomy is an important element in nurturing interest acquisition, challenge seeking, personal growth, and the wellbeing of students (Ryan and Deci, 2000; Deci et al., 2017; Tse et al., 2020). From the perspective of biological sensitivity to context theory, the underdevelopment of autonomy may increase the vulnerability of adolescents in other key areas of development, such as poorer emotion regulation, more mental health problems, and risk-taking behaviors (Earl et al., 2017; Cook et al., 2018). An individual’s development from childhood to adulthood is fraught with many risks and vulnerabilities, and key changes in the developmental trajectory of adolescence can have a critical impact on an individual’s life (Dahl et al., 2018).

Individual autonomy can only be achieved through practice, and the study of autonomy cannot be separated from social situations and the context of human beings. The report of the 19th National Congress of the Communist Party of China clearly states that Chinese socialism has entered a new era (The Xinhua News Agency, 2017). Youths are the hope of the country and the future of the nation, as well as the successors of the new era. Thus, youth work in the new era should consider their real needs and objective status quo while following the patterns of their development (Arnett, 1999). At present, the development of the Internet and the dissemination of self-media have a significant impact on the physical and mental development of adolescents (Throuvala et al., 2019; Uzun and Kilis, 2019). “Everyone is online, every day, and all the time” has become the new normal, and the Internet has become an important battlefield for adolescents (Ning and Xiao, 2019). The uniqueness of cyberspace gives a new meaning to autonomy. On the one hand, the plurality and convenience of the Internet give adolescents more space for autonomous choices (Goke et al., 2021; Rivers et al., 2021b). They can use the Internet to cultivate their awareness of information and capabilities in information integration, as well as improve their problem-solving skills (Uzun and Kilis, 2019; Sun et al., 2022). The virtuality and anonymity of the Internet can also increase adolescents’ willingness to express themselves autonomously and promote the development of their self-identity (Newland et al., 2018). On the other hand, the virtuality, anonymity, and randomness of online communications have weakened adolescents’ abilities to interact in real life, while also diminishing their responsibility and self-discipline (Throuvala et al., 2019; Ranney and Troop-Gordon, 2020). Taken together, this illustrates the inconsistency between online and offline autonomy development among adolescents (Ortiz et al., 2017; Achterhof et al., 2022), and previous studies on adolescent autonomy beyond the context of the Internet no longer fully reflect the development of autonomy among contemporary adolescents.

The existing studies on the structure of adolescent autonomy only involve offline life (Van Petegem et al., 2015; Komissarouk et al., 2017), learning (Earl et al., 2017; Mouratidis et al., 2017), parent–child relationships (Xiang and Liu, 2018; Kiang and Bhattacharjee, 2019), and other aspects (Allen and Loeb, 2015; Cook et al., 2018; Zhang et al., 2019) but have not yet considered the impact of the Internet ecological subsystem on the development of adolescent autonomy. In addition, previous studies have used top-down quantitative methods to explore the structure of adolescent autonomy, and theoretical assumptions are generated prior to conducting the study (Hmel and Pincus, 2002; Beyers et al., 2005; Bekker and Van Assen, 2006; Oğuz, 2013). This approach overlooks the contextual complexities of the era, and the uniqueness of individual development, which makes it difficult to explain the internal psychological processes of contemporary adolescents (Zhang et al., 2019). Therefore, based on grounded theory and focusing on the adolescents’ perspective, this study constructed connotations of adolescent autonomy from the bottom up through in-depth interviews and developed a questionnaire on adolescent autonomy within the context of contemporary Chinese culture. Through this process, we aimed to develop and validate an effective assessment tool that would have utility in adolescent personality development and mental health.

## Qualitative Procedure: Connotation and Theoretical Structure of Autonomy

In the present study, an exploratory mixed design was employed to conduct the questionnaire development. Mixed-methods research emphasizes logical assumptions to guide data collection and analysis, and focuses on the combination of qualitative and quantitative data, providing a more comprehensive and in-depth understanding of the research questions compared to purely quantitative or qualitative research (Yue et al., 2018; Luo et al., 2021). The first procedure of the study was based on rooted theory, valued the person’s perspective, and constructed the connotation and structure of adolescent autonomy from the bottom up through in-depth interviews.

### Participants and Interview

The purposive sampling principle of qualitative research was adopted to conduct interviews in six provinces and municipalities, including Anhui, Henan, Hubei, Guangdong, Jiangxi, and Chongqing. A total of 44 interviewees were recruited, including 40 secondary school students, two parents, and two secondary school teachers. Among them, there were 17 junior middle school students and 23 senior, 21 male students and 19 female, 22 students from urban areas and 18 from rural areas, 20 students from elite secondary schools, and 20 from regular secondary schools. The students’ ages ranged from 13 to 19 years (*M* = 16, *SD* = 1.74).

The initial interviewing team consisted of seven people, including one psychology professors, two psychology doctoral students, and four psychology master’s students. Before conducting the formal interview, the team members conducted several pre-interviews based on the compilation of relevant literature. Subsequently, the original interview outline was revised. In order to fully understand the developmental characteristics of adolescents’ autonomy, different versions of the interview outlines were prepared for students, parents, and teachers. The first part of the interview outline involved basic information about the interviewee. The second part explored the developmental characteristics of adolescent autonomy in four domains: adolescents’ lives, learning, interpersonal interactions, and Internet use. Eight to nine open-ended questions were designed for interviewees with different identities, focusing on the developmental characteristics of adolescent autonomy in cognition, behavior, and emotion. For example, the adolescent version of the question, “In what areas of life can you make your own decisions?” corresponded with the parent version question, “In what ways do you feel your child is more independent compared to when they were younger (in primary school)?” For the teacher version, this item was “How do students who are more autonomous behave in their relationships (with peers, teachers)?” Once the interview outline was finalized, the interviewing team included 14 additional psychology master’s students with interview-specific training to ensure that the interviews were conducted effectively.

### Analyses and Results

In accordance with the ethical requirements of qualitative research, the recorded interviews were converted into verbatim transcripts. Two sets of data were eliminated due to poor interview quality. Therefore, 42 transcripts were included for analysis, which were summarized and analyzed using the Consensual Qualitative Research and NVivo 11.0. The collation and analysis of the interview data were done independently by seven members of the research team; besides, a doctoral student in psychology outside the research team was invited as an auditor to avoid stereotypical thinking or major errors in the analysis process by the research team members.

The three levels of coding were carried out from the bottom up according to grounded theory, including open coding, relational coding, and core coding, in order to further complete the theoretical construction of the connotation and structure of adolescent autonomy. First of all, the researchers used open coding to uncover 25 the primary nodes related to adolescent autonomy, then counted reference points within each mode to determine the relative importance of each node. In the second step, the researchers used relational coding to tie student and parent nodes together and derive 11 secondary nodes. Following those two steps, the researchers used core coding to determine the four core concepts that would be assessed by the questionnaire. In the end, after the reference points and material source points of the core indicators were presented (see Table 1), the researchers began to extract important concepts from the nodes according to the first-hand materials in the interviews (as shown in Table 2). The connotations of the second-level indicators contained in these four major indicators were analyzed. Indicator 1 reflects adolescents’ abilities to develop goals that meet their own needs according to different situations, and proactively control their behavior to work toward their goals. Indicator 2 reflects adolescents’ abilities to rely on their own strengths, seek support, and effectively acquire, integrate, and use resources to solve problems. Indicator 3 reflects adolescents’ abilities to form and express their own opinions and feelings, and to determine their own activity goals, action plans, and evaluation criteria. Indicator 4 reflects adolescents’ abilities to possess safety awareness, distinguish between right and wrong, resist temptations, and protect themselves from external harm. Based on previous literature and the analyzed connotations, the four indicators were named autonomous regulation, autonomous problem-solving, autonomous decision-making, and autonomous protection.

**Table 1:** Indicator framework for the interview study on adolescent autonomy.

Core coding (No. of sources, no. of reference points)	Relational coding (No. of sources, no. of reference points)	Open coding (No. of sources, no. of reference points)
Autonomous regulation (42, 258)	self-control (39, 126), self-regulation (34, 84), conscious initiative (28, 48)	control (33, 73), internet dependence (30, 53), planning (31, 64), stress relief (13, 20), conscious initiative (28, 48)
Autonomous problem-solving (41, 224)	independent problem-solving (36, 130), seeking support (30, 71), resource use (17, 23)	independence (27, 50), thinking (23, 30), problem-solving (20, 38), helping others (10, 12), consulting teachers (20, 22), support from friends (19, 23), relying on parents (12, 16), resource use (17, 23)
Autonomous decision-making (40, 209)	self-determination (34, 126), positive experiences (29, 73), autonomous expression (8, 10)	self-determination (24, 45), communication with others (23, 48), independent opinion (16, 19), self-willed (11, 14), self-confidence (21, 30), feelings (20, 39), attitude (3, 4), autonomous expression (8, 10)
Autonomous protection (37, 115)	rational responsibility (31, 55), safety awareness (24, 51)	meaning interpretation (29, 42), responsibility (8, 13), self-awareness (21, 29), safety rationality (14, 22)

**Table 2:** Examples of typical viewpoints in interview materials.

Role of the interviewees	The topics	Examples of the typical viewpoints
Secondary school students	Autonomous expression	*“Express the ideas according to different occasions.”* *“Some words are more appropriate to say through WeChat or QQ chat software than face to face.”* *“Hope to express my own unique views, do not like what others say.”*
	Problem solving	*“I usually solve the problem by myself through the Internet. If I cannot solve the problem, I will ask other students, sometimes I will ask the teachers.”* *“I’ll ask my mom for school supplies and my dad for money to go out.”*
	Behavior regulation	*“I make plans for myself.”* *“Sometimes I search for the information online, but I get drawn away by other information.”* *“I cannot help playing with my phone.”*
Parents of students	Problem solving	*“My daughter’s network skills are better than mine, and she would buy gifts for me from the Internet.”* *“I usually do not have any demands for children’s housework, laundry, and cooking;”* *“When I’m busy, my child will order takeout and hail a taxi on his own, which I feel relieved about.”*
	Security defense	*“I am quite confused about the use of the Internet. I hope my children can learn more extra-curricular knowledge through the Internet, but I am afraid that my children may come into contact with bad people or things on the Internet, and I do not know how to prevent and control them.”*
	Parent–child relationship	*“I had argued with my kids, but she still told me what happened at school when she got home.”* *“I think the children still care about me. They will ask me what is wrong when seeing me unhappy.”*
Secondary school teachers	Autonomous learning	*“In class, I advocated students to study independently and let students draw their own mind maps.”* *“Nowadays, students have a wide range of knowledge. When students answer questions, they cite some examples that I do not understand. They all say that they learned by themselves on the Internet.”*
	Relationships maintaining	*“Some students often take the initiative to chat with the teacher. When there is no question to ask, they will find other topics and will take the initiative to make fun of the teacher. They are very good at watching the person’s every mood.”*
	Problem solving	*“Nowadays, few students dare not ask questions, and they have a strong sense of rights protection. If the teacher stayed in other classes for a long time during the evening self-study, they will raise opinions.”* *“Students with good grades will only listen to what they want to hear in class, will not listen to what they think they can do, and will write other papers.”*

As shown in Table 2, when the students, parents, and teachers talk about autonomy in the interviews, in addition to the conventional autonomous regulation and autonomous expression, they also cover aspects, such as network use, interpersonal relationships, problem-solving, and safety defense. This is quite different from the findings of previous studies on autonomy (Wray-Lake et al., 2010; Van Petegem et al., 2015; Alonso-Stuyck et al., 2018). Under independent decision and expression, Chinese teenagers will consider the needs of others. While they need to show their unique self-awareness, they also need to maintain contact with other important people and attach importance to achieving a negotiated balance between the two. The adolescents with high levels of autonomy know the strategies required to achieve their goals. They have open and trusting relationships with parents, peers, and teachers, and have strong social skills.

The influence of the Internet on the autonomy of the teenagers is reflected in problem-solving, behavior regulation, security defense, and autonomous learning. Thus, under the background of network culture, people have a new understanding of multiple concepts related to autonomy (Rodríguez-de-Dios et al., 2018; Throuvala et al., 2019; Ranney and Troop-Gordon, 2020) including autonomous decision-making, autonomous regulation, autonomous protection, and autonomous problem-solving. Therefore, the development of a new questionnaire on adolescent autonomy based on this connotation structure is necessary to further reveal adolescent autonomy development in the network age.

## Quantitative Procedure: Development and Validation of an Autonomy Questionnaire

### Methods

#### Participants

The quantitative stages of the study adopted cluster random sampling to distribute an online questionnaire to the students. The sample of the initial questionnaire came from 930 secondary school students in 17 cities in China. After eliminating invalid questionnaires (response time ≤ 200 s, repetitive or regular response patterns to 10 or more questions), 775 valid questionnaires remained—a valid response rate of 83.33%. The participants included 327 boys (42.19%) and 448 girls (57.81%), with 386 (49.81%) students in grade 7, 89 (11.48%) in grade 8, 141 (18.19%) in grade 9, 30 (3.87%) in grade 10, 97 (12.51%) in grade 11, and 32 (4.13%) in grade 12. The sample was randomly divided into Sample 1 and Sample 2. Exploratory factor analysis (EFA) was performed using Sample 1 (*n* = 367), and confirmatory factor analysis (CFA) was performed using Sample 2 (*n* = 408).

A third sample was used to examine the criterion validity of the Adolescent Autonomy Questionnaire resulting from the factor analysis studies. A total of three public secondary schools in Henan, Hubei, and Chongqing were selected. The classes of students were selected by cluster random sampling, respectively. Then, the offline paper questionnaire was administered and tested centrally. Researchers instructed students to fill out questionnaires for about 20 min. A total of 650 questionnaires were distributed and 614 valid questionnaires were returned—a valid response rate of 94.46%. The participants included 314 boys (51.14%) and 300 girls (48.86%), with 141 (22.96%) students in grade 7, 145 (23.62%) in grade 8, 178 (28.99%) in grade 10, and 150 (24.43%) in grade 11.

Informed consent was obtained from the school, parents, and students for all survey procedures of the study.

#### Item Development

In this study, grounded theory served as a basis for the theoretical structure of adolescent autonomy (i.e., autonomous decision-making, autonomous regulation, autonomous problem-solving, and autonomous protection). With reference to individual items from the Questionnaire on Autonomy in Middle School Students (Zou and Jia, 2008), Adolescent Independence Ability Scale (Wei, 2008), Internet Usage Self-Control Scale (Ou-Yang et al., 2013), and other instruments combined with the narrative material from our interview study, a total of 76 items were developed as a preliminary questionnaire to assess adolescent autonomy. The items covered four domains: adolescents’ lives, learning, interpersonal interactions, and Internet use. The response format for the items was a five-point scale, where 1 = “completely disagree” and 5 = “completely agree.” The responses for all subscale items were summed to provide a subscale score, and subscale scores were summed to provide a total scale score.

#### Criterion-Related Validity Measures

We used two measures to examine criterion-related validity: The Personal Growth Initiative Scale-II (PGIS-II) and the Adolescence Ego Identity Crisis Scale (AEICS). The PGIS-II was developed by Robitschek et al. in 2012 (Guo, 2018). The scale consists of 16 items and includes four dimensions: readiness for change, planfulness, using resources, and intentional behavior. Responses use a six-point Likert scale (0 = “completely disagree,” 5 = “completely agree”), with higher scale scores indicating higher levels of personal growth initiative. In this study, the internal consistency reliability coefficient was 0.929.

The AEICS was developed by Byrd in 1972 (Jiang, 1991). It includes the Positive and Negative subscales and consists of 28 items. Item responses use a five-point Likert scale (1 = “completely disagree,” 5 = “completely agree”), and the Positive subscale items are reverse scored. Subscale scores are summed to obtain a total score, with higher scores indicating higher levels of identity crisis and lower levels of ego identity. In the present study, the internal consistency reliability coefficient was 0.879.

#### Statistical Analysis

SPSS 24.0 was used for the item analysis, correlation analysis, reliability analysis, and EFA. Mplus 7.0 was used for the CFA.

### Results

#### Item Analysis

Item analysis was performed on the preliminary test sample (*n* = 775), including independent samples *t*-test and overall item correlations for both high- and low-scoring groups. The results showed that all items reached a significance level of *p* < 0.001 in the difference between the high and low groups. However, four items were deleted because they had an overall item correlation of less than 0.40.

#### Exploratory Factor Analysis

Exploratory factor analysis was performed on the remaining 72 items of the preliminary questionnaire using Sample 1 (*n* = 367). The KMO value was 0.955 and the Bartlett sphericity test *χ*^2^ = 15403.43 (*df* = 2,556, *p* < 0.001), which indicated that the data were suitable for EFA. Factors were extracted using principal component analysis, and the factor structure was determined based on the eigenvalues-greater-than-1 rule and with reference to the scree plot. In the factor exploration process, the scale items were selected based on the following criteria: (i) Screening based on communality. We removed items with the communality of less than 0.30 to ensure that each item contributed significantly to the extracted common factors. (ii) Screening based on the item loadings. A high loading value indicates that the item is closely associated with the common factor. In this study, items with loading values greater than 0.40 were retained. (iii) Cross-loading values. Items with high loadings on two or more factors were difficult to categorize and were removed. (iv) The number of items per factor was not less than 3. (v) Deleting items that are classified as not applicable to theory or logic. The exploratory factor analysis should be redone after each deletion, generally starting with the lowest loaded item (Fabrigar et al., 1999). After several explorations, a three-factor structure with 16 items was retained. However, the scree plot indicated that the curve only leveled off when four factors were extracted. Furthermore, we took into account the theoretical construction of autonomy based on the qualitative interview results and fixed the number of common factors to be extracted at four. The results showed a clear four-factor structure, of which autonomous decision-making included five items, autonomous protection included four items, autonomous regulation included four items, and autonomous problem-solving included three items. The total variance explained was 62.54%. The factor loadings and communality for the 16 items are shown in Table 3.

**Table 3:** Results of the exploratory factor analysis on the Adolescent Autonomy Questionnaire.

Item	Factor loadings				Communality
	F1	F2	F3	F4	
F1: Autonomous decision-making					
5.I dare to express my own novel and different opinions.	0.730				0.591
12.I know what I need, like, and am good at.	0.713				0.614
8.I feel comfortable expressing my opinions and feelings when communicating with others.	0.670				0.559
1.I can make up my own mind at critical times.	0.668				0.528
15.I feel confident in the decisions I have made.	0.653				0.536
F2: Autonomous protection					
10.I can decisively reject harmful invitation messages online.		0.794			0.707
14.I can consciously resist the intrusion of bad information on the Internet.		0.788			0.705
2.I do not randomly scan QR codes on advertising.		0.740			0.609
6.I do not add random strangers as friends on social media.		0.686			0.644
F3: Autonomous regulation					
3.I am able to complete my studies as planned.			0.763		0.654
9.In addition to completing the homework assigned by my teacher, I take the initiative to do other exercises or reviews.			0.749		0.631
13.I allocate study times to different learning tasks in a reasonable and effective manner.			0.683		0.692
7.I am able to make my own arrangements for my studies without parental supervision.			0.662		0.564
F4: Autonomous problem-solving					
16.I usually gather information from a variety of sources to solve problems I encounter.				0.761	0.673
11.I am able to understand and use Internet resources well.				0.710	0.685
4.I can use surrounding objects to save myself in case of accidents or injuries.				0.644	0.612

#### Confirmatory Factor Analysis

**Figure 1:**
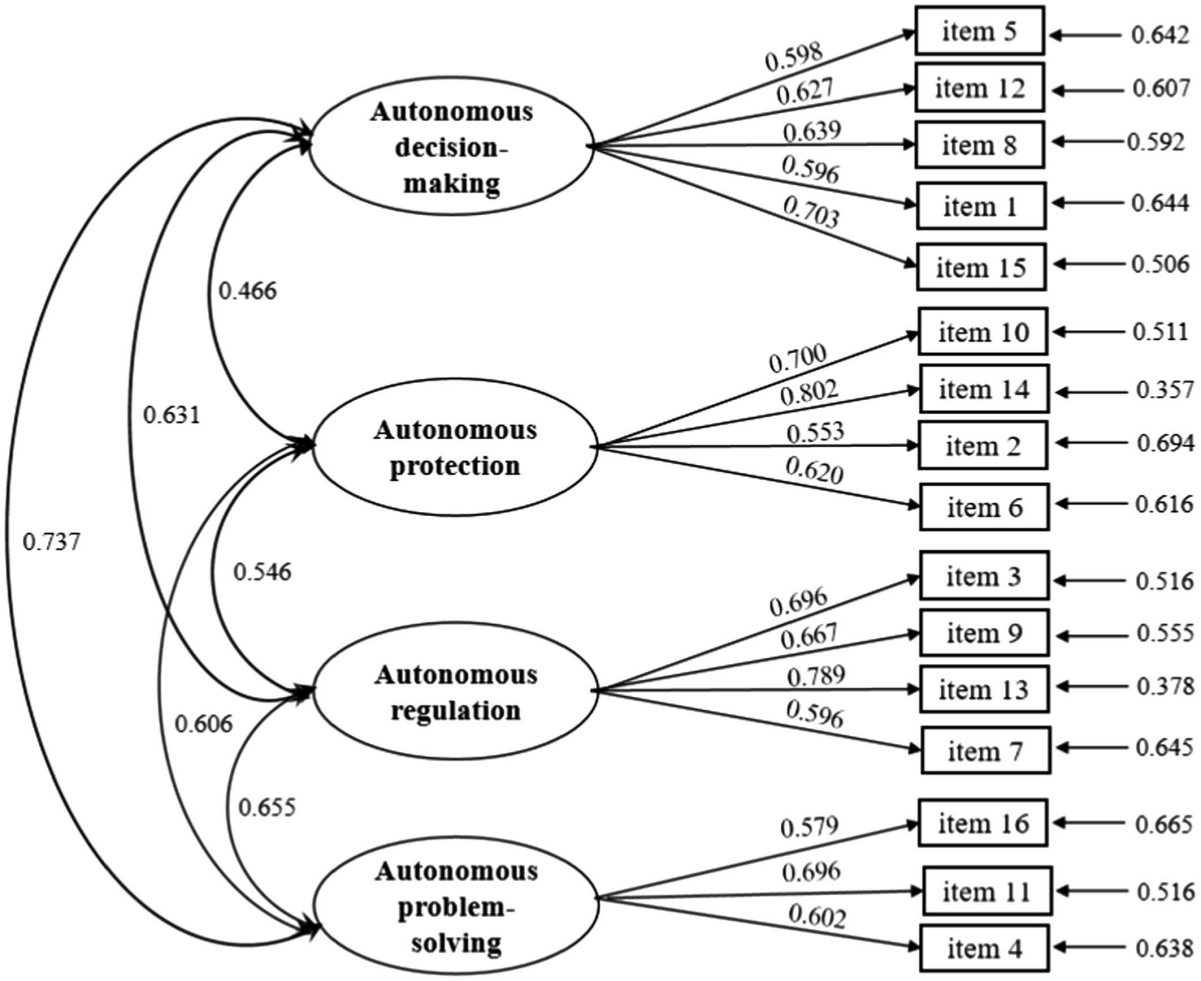
Standardized parameters of the four-factor model of the Adolescent Autonomy Questionnaire.

Confirmatory factor analysis was performed on the structure of the Adolescent Autonomy Questionnaire using Sample 2 (*n* = 408). In order to examine whether the four-factor model was superior, we compared it to three other possible models. The three competitive models were as: three-factor model, domain model, and second-order model. The three-factor model shared the same items as the four-factor model and included autonomous decision-making, autonomous protection, and autonomous regulation. The domain model was discovered during our exploratory factor analysis, which consisted of four factors with a total of 19 items. These four factors represent the four domains of adolescent autonomy in interpersonal expression, learning regulation, Internet use, and independent living, which are consistent with the four domains of autonomy constructed in our interview outline. In addition, considering that the second-order model may be more concise than the first-order model in terms of measurement statistics and structural interpretation, we tried to construct a second-order model based on the first-order four-factor model. The fit indices comparing the structure of the Adolescent Autonomy Questionnaire with the competing models are shown in Table 4. The fit indices of the four-factor model were better than those of the three-factor model and the second-order model. Meanwhile, the fit indices of the four-factor model were relatively similar to those of the domain model, and both met the fit criteria for model validation. However, given the theoretical conception of autonomy and the operability of practical interventions, we decided to use the four-factor model as the structural model for the Adolescent Autonomy Questionnaire. The figure of standardized parameters of the four-factor model of the Adolescent Autonomy Questionnaire can be found in Figure 1.

**Table 4:** Comparison of fit indices between the structure of the Adolescent Autonomy Questionnaire and competing models.

	*χ* ^2^	*df*	*χ* ^2^ */df*	CFI	TLI	RMSEA	SRMR
Four-factor model	229.32	98	2.340	0.949	0.938	0.042	0.046
Three-factor model	278.97	101	2.762	0.927	0.913	0.050	0.054
Domain model	326.00	146	2.233	0.951	0.943	0.042	0.046
Second-order model	235.07	100	2.350	0.948	0.937	0.042	0.048

#### Reliability Analysis

Internal consistency reliability was evaluated with Cronbach’s alpha. The coefficients for the subscales were as follows: autonomous decision-making, 0.800; autonomous protection, 0.810; autonomous regulation, 0.793; and autonomous problem-solving, 0.734. The Cronbach’s alpha for the total scale was 0.894.

#### Structural Validity

The results of the CFA indicated that the structure of the Adolescent Autonomy Questionnaire was reasonable. Additionally, each subscale of the questionnaire was closely correlated with the total score (Table 5), and the correlations between each subscale and the total score (*r* = 0.71–0.81) were higher than those between the subscales (*r* = 0.36–0.54). These results provide further support for the structural validity of the Adolescent Autonomy Questionnaire.

**Table 5:** Correlations between the Adolescent Autonomy Questionnaire total scale score and each subscale score.

Scales	F1	F2	F3	F4	Total scale score
F1. Autonomous decision-making	–				
F2. Autonomous regulation	0.477^**^	–			
F3. Autonomous protection	0.363^**^	0.435^**^	–		
F4. Autonomous problem-solving	0.537^**^	0.456^**^	0.423^**^	–	
Total scale score	0.805^**^	0.792^**^	0.711^**^	0.745^**^	–

** *p < 0.01*.

#### Criterion-Related Validity

Criterion validity analysis was performed using a third group of participants (*n* = 614). The Adolescent Autonomy Questionnaire was correlated with the PGIS-II and AEICS (Table 6). The results of correlation analysis indicated there was a significant positive correlation between the Adolescent Autonomy Questionnaire and the PGIS-II, and a significant negative correlation with the AEICS. These findings further corroborate the validity of the Adolescent Autonomy Questionnaire.

**Table 6:** Correlation analysis of the Adolescent Autonomy Questionnaire total score and subscale scores with the criterion scales.

Scales	Autonomous decision-making	Autonomous regulation	Autonomous protection	Autonomous problem-solving	Total scale score
PGIS-II	0.483^**^	0.468^**^	0.338^**^	0.468^**^	0.592^**^
AEICS	−0.651^**^	−0.481^**^	−0.301^**^	−0.485^**^	−0.654^**^

*PGIS-II, Personal Growth Initiative Scale-II; AEICS, Adolescence Ego Identity Crisis Scale*.

** *p < 0.01*.

## Discussion

At present, autonomy is widely regarded as a type of personality trait, competency, or ability. The different definitions of autonomy reflect the different focal points of scholars in their research on autonomy, while the diversity of definitions also reflects its rich connotations. In understanding autonomy, its connotations should be neither too narrow nor too generalized (Chai, 2015). Given our considerations of adolescent personality development patterns and the potential for intervening in this process, we viewed adolescent autonomy as an ability that can be developed. Therefore, the concept of autonomy discussed in this study refers to the ability of an individual, as a behavioral actor, to rely on their own strength to respond effectively to complex and changing environments and promote self-development. The autonomy of contemporary Chinese adolescents refers to the ability of adolescents, as the initiators of action, to make decisions and perform actions in accordance with their true intentions in their daily life, learning, interpersonal communication, and Internet use. This is achieved through the regulation of their cognitions, emotions, and behaviors which leads to effective problem-solving, self-protection, and promotion of continuous self-growth. This definition reflects the main domains and core elements in adolescent autonomy development, as well as the local cultural and contemporaneous characteristics of this concept.

In order to ensure that the indicators of the Adolescent Autonomy Questionnaire met psychometric requirements, we strictly adhered to the following steps in preparing the questionnaire. First, the theoretical connotations and structure of adolescent autonomy in contemporary China were clarified based on grounded theory. Second, factor analysis was performed to verify the rationale of the structural components of autonomy derived from the qualitative portion of our study. A total of 16 items and four factors were confirmed to form the final version of the Adolescent Autonomy Questionnaire. The four factors were labeled autonomous decision-making, autonomous regulation, autonomous protection, and autonomous problem-solving.

Autonomous decision-making represents adolescents’ shift from a state of dependence on others to a state of self-reliance, in which they can make better decisions by assessing the negative consequences of their decisions. Chinese culture attaches great importance to close interpersonal relationships and interdependence (Fuligni, 1998). Thus, adolescents’ autonomous decision-making requires them to achieve a negotiated balance between demonstrating a unique sense of self and maintaining contact with the other significant people in their lives. Highly autonomous adolescents know what strategies to use to achieve their goals have open and trusting relationships with their parents and peers, and possess strong social skills. Therefore, we need to further develop the self-approval of adolescents and encourage them to act according to their own personal values and interests (Dahl et al., 2018).

Autonomous regulation means that adolescents are able to make plans, set goals, identify and evaluate existing and needed resources, balance resources to meet different goals, learn from past behaviors, anticipate future outcomes, monitor the process, and make necessary adjustments in the execution of the plan. Adolescents’ autonomous regulation involves multiple cognitive, emotional, and behavioral processes, such as awareness, reflection, responsibility, and self-discipline. However, contemporary adolescents seem to have become passive consumers of technology. The starting point for the creation of intelligent technology by humans originated from self-liberation, but when artificial intelligence arrives, human freedom and liberation may face an unprecedented test (Ranney and Troop-Gordon, 2020). As adolescents’ dependence on the Internet continues to deepen, they have created an “information cocoon” for themselves, causing them to refuse communication with the outside world and possess an increasingly narrow worldview (Yang, 2019). Faced with these circumstances, the adolescents of today will need to improve their Internet literacy and judgment ability, enhance their ability to process information in online environments, and strengthen their offline social interactions and communication.

In the past, the protection system for adolescents mainly relied on family, school, and society. However, in the current self-media era, factors, such as poor Internet supervision and the abuse of Internet technology, have led to the frequent occurrences of online fraud, online rumors, and cyberbullying. These incidents pose new threats to the healthy development of adolescents, and many parents believe that they do not have sufficient ability to protect their children from the dangers of the Internet. Parents often face the dilemma of wanting their children to use computers and the Internet for learning, but also worrying about their children’s exposure to undesirable information. This problem is further compounded by their limited knowledge on how to monitor or protect their children’s Internet use (Bynum and Kotchick, 2006). Therefore, adolescents need to strengthen their ability for autonomous protection, establish correct concepts of Internet safety, distinguish between good and bad information on the Internet, and assume their responsibility as main participants in self-media.

Autonomous problem-solving is one of the core autonomous development indicators of greatest concern to the state and parents of middle school students (Hu and Lin, 2018). It reflects an individual’s ability to comprehensively use the environment and their internal resources to effectively solve problems encountered in real social and life environments, and to achieve self-development. In addition, planning, interpersonal communication, support seeking, and resource integration are the core skills needed by adolescents in the process of solving daily problems. Information technology has given rise to opportunities for humans to enhance their problem-solving skills. It makes problem-solving faster and more convenient, while also enhancing the need for problem-solving skills. In the online environment created through modern information technology, the problem-solving ability of adolescents does not simply refer to the mastery of knowledge and problem-solving skills but is more concerned with their problem-solving process. Students become “inquirers” of learning, use Internet resources to find information, discover problems, explore new knowledge, perform self-assessment, and achieve autonomous innovation. This will significantly stimulate the enthusiasm and initiative of adolescents to solve problems independently and attain autonomy in a real sense (Hu and Lin, 2018).

### Implications for Research and Practice

The existence of diverse selves in cyberspace has brought unprecedented challenges to the autonomy development of humans (Boniel-Nissim et al., 2022; Hayes, 2022). The diverse self-expression (Throuvala et al., 2019; Duvenage et al., 2020), the consistency between online and offline (Rivers et al., 2021a; Achterhof et al., 2022), the correction of alienated selves (Coyne et al., 2019), and the avoidance of various false selves (Goke et al., 2021; Rivers et al., 2021b) have become new issues of self-development faced by the adolescents in the Internet era. In this study, the literature on autonomy worldwide was reviewed and compared to further explore adolescents’ autonomy in the context of network culture. This somewhat expands the measurement dimensions of autonomy. Further, it compensates for the little existing research on the development of autonomy from the perspective of social reality and adolescents themselves.

The Adolescent Autonomy Scale developed in this study can provide useful guidance for both the cultivation of adolescent autonomy and the correction of social maladjustment problems. It lays a scientific foundation for the implementation of subjectivity education and the cultivation of healthy personality in families and schools. Regarding the educational goals of adolescent autonomy, the pros and cons of the development of adolescent autonomy cannot be treated solely from individual characteristics or psychological state without the consideration of the more crucial dependence relationship behind the autonomy. At present, how the Internet promotes or inhibits the development of adolescent autonomy is unclear, while attention should be paid to the development of adolescent autonomy in the context of network culture (Duvenage et al., 2020; LaTour and Noel, 2021). Students, teachers, or parents should not rely too much on artificial intelligence and weaken their own subjective functions. From the viewpoint of the educational content of adolescent autonomy, autonomous decision-making, autonomous regulation, autonomous protection, and autonomous problem-solving are the core contents of adolescent autonomy development nowadays and balance the relationship between self and others, individuals, and society. Independence and integration remain the major topics of adolescent autonomy development. From the perspective of the educational channels of adolescent autonomy, besides family and school, we should also vigorously promote the educational function of the network platform for adolescent autonomy. Educational technologists should develop potential ways to enhance youth autonomy from the Internet (Chen et al., 2017), such as building an online platform for independent learning and living to facilitate cooperative communication, resource integration, and problem-solving for adolescents. The network regulatory workers should introduce network regulatory measures with technical support, strengthen the network cultural information and network security supervision, and create a green and civilized network cultural environment for the healthy development of the adolescents.

### Limitations and Future Research

Although this study has constructed an adolescent autonomy measurement index system and compiled its questionnaire, some problems need to be explored in-depth in the next phase of the study. First, the selections of the subject samples were not ideal. There was a lack of participants from the 9th and 12th grades due to COVID-19 and academic pressure; therefore, the study did not cover the whole secondary school stage of adolescents. This study only involved Chinese adolescents with a collectivist culture background. This cannot be compared with a Western individual culture background, which prevents it from highlighting the uniqueness of Chinese adolescents’ autonomy development. Individuals in different situations have different needs for autonomy, and their autonomy levels also reflect different meanings. For example, the autonomy of adolescents in the context of Western culture generally develops in a straight line (Oudekerk et al., 2015), while the autonomy of Chinese adolescents generally moves in a spiral from elementary school to university. The level of autonomous development of high school students is lower than that of junior high school students (Qin et al., 2009; Kiang and Bhattacharjee, 2019). This may be induced by the huge pressure of the Chinese college entrance examination and the influence of factors, such as filial piety in traditional Chinese culture (Lee et al., 2010; Sun et al., 2019). Therefore, participants from all the grades in secondary school and different cultural backgrounds should be selected in future studies. Furthermore, the vertical development of youth autonomy in junior high school, high school, and university should be stressed to objectively understand the autonomy development of local youth.

Additionally, the present study relied mainly on participants’ self-reported or hypothetical situational responses; therefore, their association with actual behaviors needs to be repeated. More objective experiments or longitudinal follow-up studies can be employed in future research to better understand the trends and patterns of adolescent autonomy development in an online environment.

Finally, the present study lacked the discussion of the internal mechanism and various influencing factors in the formation of adolescent autonomy. In future research, the moderating role of certain critical protective and individual factors in the relationship between risk or environmental factors and adolescent autonomy development may be explored. Moreover, the authority control theory posits that parental authority control and autonomy support are essential factors influencing the development of adolescents’ autonomy (Xiang and Liu, 2018). However, adolescents may have problems with the development of internal control if parents provide adolescents with the right to autonomy too early and excessively (Van Petegem et al., 2015). Then, when will adolescents gain a sense of autonomy, what level of autonomy is more suitable for them? Follow-up research can reveal the deep-seated mechanism of adolescents’ autonomous development trajectory from these perspectives. The development of intervention programs to increase adolescent autonomy is the core value of the reality of autonomy research.

## Data Availability Statement

All data included in this study are available upon request by contact with the first author (YL: lypsy@foxmail.com).

## Ethics Statement

The studies involving human participants were reviewed and approved by Research Ethics Committee of Central China Normal University. Written informed consent to participate in this study was provided by the participants’ legal guardian/next of kin.

## Author Contributions

YL: data analysis, investigation, and writing—original draft. HC and Y-lZ: writing—review and editing. L-lW: formal analysis and investigation. C-yF: design, supervision, and funding acquisition. All authors contributed to the article and approved the submitted version.

## Funding

This work was supported by the Special Monitoring Project of the China Collaborative Innovation Center of Assessment for Basic Education Quality, Beijing Normal University (grant number 2019-04-009-BZPK01).

## Conflict of Interest

The authors declare that the research was conducted in the absence of any commercial or financial relationships that could be construed as a potential conflict of interest.

## Publisher’s Note

All claims expressed in this article are solely those of the authors and do not necessarily represent those of their affiliated organizations, or those of the publisher, the editors and the reviewers. Any product that may be evaluated in this article, or claim that may be made by its manufacturer, is not guaranteed or endorsed by the publisher.

## References

[ref1] Achterhof R. Kirtley O. J. Schneider M. Hagemann N. Hermans K. S. F. M. Hiekkaranta A. P.. (2022). Adolescents’ real-time social and affective experiences of online and face-to-face interactions. Comput. Hum. Behav. 129:107159. doi: 10.1016/j.chb.2021.107159

[ref2] Allen J. P. Loeb E. L. (2015). The autonomy-connection challenge in adolescent peer relationships. Child Dev. Perspect. 9, 101–105. doi: 10.1111/cdep.12111, PMID: 25937829 PMC4414255

[ref3] Alonso-Stuyck P. Zacarés J. J. Ferreres A. (2018). Emotional separation, autonomy in decision-making, and psychosocial adjustment in adolescence: a proposed typology. J. Child Fam. Stud. 27, 1373–1383. doi: 10.1007/s10826-017-0980-5

[ref4] Anderson R. A. Worthington L. Anderson W. T. Jennings G. (1994). The development of an autonomy scale. Contemp. Fam. Ther. 16, 329–345. doi: 10.1007/BF02196884

[ref503] Arnett J. J. (1999). Adolescent storm and stress, reconsidered. Am. Psychol. 54, 317–326. doi: 10.1037/0003-066X.54.5.317, PMID: 10354802

[ref5] Bekker M. H. J. Van Assen M. A. L. M. (2006). A short form of the autonomy scale: properties of the autonomy-connectedness scale (ACS–30). J. Pers. Assess. 86, 51–60. doi: 10.1207/s15327752jpa8601_07, PMID: 16436020

[ref504] Beyers W. Goossens L. Van Calster B. Duriez B. (2005). An alternative substantive factor structure of the emotional autonomy scale. Eur. Psychol. Assess. 21, 147–155. doi: 10.1027/1015-5759.21.3.147

[ref6] Beyers W. Goossens L. Vansant I. Moors E. (2003). A structural model of autonomy in middle and late adolescence: connectedness, separation, detachment, and agency. J. Youth Adolesc. 32, 351–365. doi: 10.1023/A:1024922031510

[ref7] Boniel-Nissim M. van den Eijnden R. J. J. M. Furstova J. Marino C. Lahti H. Inchley J.. (2022). International perspectives on social media use among adolescents: implications for mental and social well-being and substance use. Comput. Hum. Behav. 129, 107144. doi: 10.1016/j.chb.2021.107144

[ref8] Bynum M. S. Kotchick B. A. (2006). Mother-adolescent relationship quality and autonomy as predictors of psychosocial adjustment among African American adolescents. J. Child Fam. Stud. 15, 528–541. doi: 10.1007/s10826-006-9035-z

[ref9] Campbell D. G. Kwon P. Reff R. C. Williams M. G. (2003). Sociotropy and autonomy: an examination of interpersonal and work adjustment. J. Pers. Assess. 80, 206–207. doi: 10.1207/S15327752JPA8002_09, PMID: 12700023

[ref10] Chai Y. J. (2015). Student learning autonomy: connotation, features and mechanism. Contemp. Educ. Cult. 7, 38–44. doi: 10.13749/j.cnki.cn62-1202/g4.2015.04.008

[ref11] Charry C. Goig R. Martínez I. (2020). Psychological well-being and youth autonomy: comparative analysis of Spain and Colombia. Front. Psychol. 11:564232. doi: 10.3389/fpsyg.2020.564232, PMID: 33101134 PMC7545337

[ref12] Chen H. Beaudoin C. E. Hong T. (2017). Securing online privacy: an empirical test on internet scam victimization, online privacy concerns, and privacy protection behaviors. Comput. Hum. Behav. 70, 291–302. doi: 10.1016/j.chb.2017.01.003

[ref13] Cook E. C. Wilkinson K. Stroud L. R. (2018). The role of stress response in the association between autonomy and adjustment in adolescents. Physiol. Behav. 189, 40–49. doi: 10.1016/j.physbeh.2018.02.049, PMID: 29501557 PMC5882503

[ref14] Coyne S. M. Stockdale L. Summers K. (2019). Problematic cell phone use, depression, anxiety, and self-regulation: evidence from a three year longitudinal study from adolescence to emerging adulthood. Comput. Hum. Behav. 96, 78–84. doi: 10.1016/j.chb.2019.02.014

[ref15] Dahl R. E. Allen N. B. Wilbrecht L. Suleiman A. B. (2018). Importance of investing in adolescence from a developmental science perspective. Nature 554, 441–450. doi: 10.1038/nature25770, PMID: 29469094

[ref16] Deci E. L. Olafsen A. H. Ryan R. M. (2017). Self-determination theory in work organizations: the state of a science. Annu. Rev. Organ. Psychol. Organ. Behav. 4, 19–43. doi: 10.1146/annurev-orgpsych-032516-113108

[ref17] Duvenage M. Correia H. Uink B. Barber B. L. Donovan C. L. Modecki K. L. (2020). Technology can sting when reality bites: adolescents’ frequent online coping is ineffective with momentary stress. Comput. Hum. Behav. 102, 248–259. doi: 10.1016/j.chb.2019.08.024

[ref18] Earl S. R. Taylor I. M. Meijen C. Passfield L. (2017). Autonomy and competence frustration in young adolescent classrooms: different associations with active and passive disengagement. Learn. Instr. 49, 32–40. doi: 10.1016/j.learninstruc.2016.12.001

[ref19] Fabrigar L. R. Wegener D. T. MacCallum R. C. Strahan E. J. (1999). Evaluating the use of exploratory factor analysis in psychological research. Psych. Meth. 4, 272–299. doi: 10.1037/1082-989X.4.3.272

[ref20] Fuligni A. J. (1998). Authority, autonomy, and parent-adolescent conflict and cohesion: a study of adolescents from Mexican, Chinese, Filipino, and European backgrounds, Chinese. Filipino. Dev. Psychol. 34, 782–792. doi: 10.1037//0012-1649.34.4.782, PMID: 9681270

[ref21] Goke R. Berndt M. Rocker K. (2021). Classroom culture when students are reluctant to learn online: student dissent behaviors explained by their self-efficacy, control of learning, and intrinsic motivation. Front. Commun. 6:641956.doi: 10.3389/fcomm.2021.641956

[ref501] Guo J. C. (2018). Research on the development characteristics, influencing factors and mechanism of adolescent students’ personal growth initiative. doctoral dissertation. Fujian, Fuzhou: Fujian Normal University. doi: 10.27019/d.cnki.gfjsu.2018.000024, PMID:

[ref23] Hayes B. James A. Barn R. Watling D. (2022). Children’s risk and benefit behaviours on social networking sites. Comput. Hum. Behav. 130:107147. doi: 10.1016/j.chb.2021.107147

[ref24] Hill J. P. Holmbeck G. N. (1986). Attachment and autonomy during adolescence. Ann. Child Dev. 11, 145–189.

[ref502] Hmel B. A. Pincus A. L. (2002). The meaning of autonomy: on and beyond the interpersonal circumplex. J. Pers. 70, 277–310. doi: 10.1111/1467-6494.05006 12049162

[ref25] Hu D. R. Lin Y. (2018). What kinds of core competencies should primary and secondary students have? From parents’ views. Educ. Res. Exp. 2018, 65–73.

[ref26] Jiang N. F. (1991). Adolescent Self-Integration and Education (Taipei: Fuwen Publishing House).

[ref27] Keller H. (2012). Autonomy and relatedness revisited: cultural manifestations of universal human needs. Child Dev. Perspect. 6, 12–18. doi: 10.1111/j.1750-8606.2011.00208.x

[ref28] Kiang L. Bhattacharjee K. (2019). Developmental change and correlates of autonomy in Asian American adolescents. J. Youth Adolesc. 48, 410–421. doi: 10.1007/s10964-018-0909-3, PMID: 30088130

[ref29] Komissarouk S. Harpaz G. Nadler A. (2017). Dispositional differences in seeking autonomy- or dependency-oriented help: conceptual development and scale validation. Pers. Individ. Dif. 108, 103–112. doi: 10.1016/j.paid.2016.12.019

[ref30] LaTour K. A. Noel H. N. (2021). Self-directed learning online: An opportunity to binge. J. Mark. Edu. 43, 174–188. doi: 10.1177/0273475320987295

[ref31] Lee C. T. Beckert T. E. Goodrich T. R. (2010). The relationship between individualistic, collectivistic, and transitional cultural value orientations and adolescents’ autonomy and identity status. J. Youth Adolesc. 39, 882–893. doi: 10.1007/s10964-009-9430-z, PMID: 20596816

[ref32] Lin C. D. (2017). To construct sinicized core competencies and values for student development. J. Beijing Norm. Univ. (Soc. Sci.) 66–73. doi: 10.3969/j.issn.1002-0209.2017.01.006

[ref33] Luo L. Snyder P. Qiu Y. Huggins-Manley A. C. Hong X. (2021). Development and validation of a questionnaire to measure Chinese preschool teachers’ implementation of social-emotional practices. Front. Psychol. 12:699334. doi: 10.3389/fpsyg.2021.699334, PMID: 34566776 PMC8460858

[ref34] Mouratidis A. Michou A. Vassiou A. (2017). Adolescents’ autonomous functioning and implicit theories of ability as predictors of their school achievement and week-to-week study regulation and well-being. Contemp. Educ. Psychol. 48, 56–66. doi: 10.1016/j.cedpsych.2016.09.001

[ref35] Newland L. A. Mourlam D. Strouse G. (2018). A phenomenological exploration of the role of digital technology and media in children’s subjective well-being. Child Ind. Res. 11, 1563–1583. doi: 10.1007/s12187-017-9498-z

[ref36] Ning Q. Xiao G. R. (2019). Jinping’s youth work thoughts in the new era. Contemp. Youth Res. 1, 26–32. doi: 10.3969/j.issn.1006-1789.2019.01.004

[ref37] Noom M. J. Deković M. Meeus W. (2001). Conceptual analysis and measurement of adolescent autonomy. J. Youth Adolesc. 30, 577–595. doi: 10.1023/A:1010400721676

[ref505] Oğuz A. (2013). Developing a scale for learner autonomy support. Educ. Sci: Theo. Pra. 13:2187. doi: 10.12738/estp.2013.4.1870, PMID: 9681270

[ref38] Ortiz J. Chang S. Chih W. Wang C. (2017). The contradiction between self-protection and self-presentation on knowledge sharing behavior. Comput. Hum. Behav. 76, 406–416. doi: 10.1016/j.chb.2017.07.031

[ref39] Oudekerk B. A. Allen J. P. Hessel E. T. Molloy L. E. (2015). The cascading development of autonomy and relatedness from adolescence to adulthood. Child Dev. 86, 472–485. doi: 10.1111/cdev.12313, PMID: 25345623 PMC4376599

[ref40] Ou-Yang Y. Zhang D. Y. Wu M. X. (2013). Development of the internet usage self-control scale for college students. Chin. Ment. Health J. 2013, 54–58. doi: 10.3969/j.issn.1000-6729.2013.01.010

[ref41] Qin L. Pomerantz E. M. Wang Q. (2009). Are gains in decision-making autonomy during early adolescence beneficial for emotional functioning? The case of the United States and China. Child Dev. 80, 1705–1721. doi: 10.1111/j.1467-8624.2009.01363.x, PMID: 19930347

[ref42] Ranney J. D. Troop-Gordon W. (2020). The role of popularity and digital self-monitoring in adolescents’ cyberbehaviors and cybervictimization. Comput. Hum. Behav. 102, 293–302. doi: 10.1016/j.chb.2019.08.023

[ref43] Rivers D. J. Nakamura M. Vallance M. (2021a). Online self-regulated learning and achievement in the era of change. J. Educ. Comput. Res. doi: 10.1177/07356331211025108 (in press)

[ref44] Rivers D. J. Vallance M. Nakamura M. (2021b). Metacognitive knowledge and the self as socially distanced online learner: a virtual reality assisted analysis of academic self-concept. J. Educ. Technol. Syst. 50, 87–111. doi: 10.1177/0047239521999779

[ref45] Robitschek C. Ashton M. W. Spering C. C. Geiger N. Byers D. Schotts G. C.. (2012). Development and psychometric evaluation of the personal growth initiative scale-II. J. Couns. Psychol. 59, 274–287. doi: 10.1037/a0027310, PMID: 22352950

[ref46] Rodríguez-de-Dios I. van Oosten J. M. F. Igartua J. (2018). A study of the relationship between parental mediation and adolescents’ digital skills, online risks and online opportunities. Comput. Hum. Behav. 82, 186–198. doi: 10.1016/j.chb.2018.01.012

[ref507] Ryan R. M. Deci E. L. (1985). The “third selective paradigm” and the role of human motivation in cultural and biological selection: a response to Csikszentmihalyi and Massimini. New Ideas Psychol. 3, 259–264. doi: 10.1016/0732-118X(85)90020-0, PMID: 11392867

[ref47] Ryan R. M. Deci E. L. (2000). Self-determination theory and the facilitation of intrinsic motivation, social development, and well-being. Am. Psychol. 55, 68–78. doi: 10.1037//0003-066x.55.1.68, PMID: 11392867

[ref48] Ryan R. M. Deci E. L. (2006). Self-regulation and the problem of human autonomy: does psychology need choice, self-determination, and will? J. Pers. 74, 1557–1586. doi: 10.1111/j.1467-6494.2006.00420.x, PMID: 17083658

[ref49] Steinberg L. Silverberg S. B. (1986). The vicissitudes of autonomy in early adolescence. Child Dev. 57, 841–851. doi: 10.1111/j.1467-8624.1986.tb00250.x, PMID: 3757604

[ref50] Sun P. Fan X. Sun Y. Jiang H. Wang L. (2019). Relations between dual filial piety and life satisfaction: the mediating roles of individuating autonomy and relating autonomy. Front. Psychol. 10:2549. doi: 10.3389/fpsyg.2019.02549, PMID: 31849735 PMC6896836

[ref51] Sun C. Shute V. J. Stewart A. E. B. Beck-White Q. Reinhardt C. R. Zhou G.. (2022). The relationship between collaborative problem solving behaviors and solution outcomes in a game-based learning environment. Comput. Hum. Behav. 128:107120. doi: 10.1016/j.chb.2021.107120

[ref52] The Xinhua News Agency (2017). Securing a Decisive Victory in building a Moderately prosperous Society in All respects and Striving for the Great Victory of socialism with Chinese characteristics for a New Era—Report at the 19th National Congress of the Communist Party of China. Available at: http://www.gov.cn/zhuanti/2017-10/27/content_5234876.htm (Accessed October 27, 2017).

[ref53] Throuvala M. A. Griffiths M. D. Rennoldson M. Kuss D. J. (2019). Motivational processes and dysfunctional mechanisms of social media use among adolescents: a qualitative focus group study. Comput. Hum. Behav. 93, 164–175. doi: 10.1016/j.chb.2018.12.012

[ref54] Tse D. C. K. Lau V. W. Perlman R. McLaughlin M. (2020). The development and validation of the autotelic personality questionnaire. J. Pers. Assess. 102, 88–101. doi: 10.1080/00223891.2018.1491855, PMID: 30183366

[ref55] Uzun A. M. Kilis S. (2019). Does persistent involvement in media and technology lead to lower academic performance? Evaluating media and technology use in relation to multitasking, self-regulation and academic performance. Comput. Hum. Behav. 90, 196–203. doi: 10.1016/j.chb.2018.08.045

[ref56] Van Petegem S. Vansteenkiste M. Soenens B. Beyers W. Aelterman N. (2015). Examining the longitudinal association between oppositional defiance and autonomy in adolescence. Dev. Psychol. 51, 67–74. doi: 10.1037/a0038374, PMID: 25419798

[ref57] Wei W. (2008). The Development of Adolescents’ Independent Ability Scale and the Research on the Current Situation. Master’s Thesis. Fujian (Fuzhou): Fujian Normal University.

[ref58] Wray-Lake L. Crouter A. C. McHale S. M. (2010). Developmental patterns in decision-making autonomy across middle childhood and adolescence: European American parents’ perspectives. Child Dev. 81, 636–651. doi: 10.1111/j.1467-8624.2009.01420.x, PMID: 20438465 PMC2864944

[ref59] Xia L. X. Huang X. T. (2012). The difference between independent personality in China and independent personality in the west. J. Southwest Univ. 38, 38–44. doi: 10.3969/j.issn.1673-9841.2012.01.006

[ref60] Xiang S. Liu Y. (2018). Understanding the joint effects of perceived parental psychological control and insecure attachment styles: a differentiated approach to adolescent autonomy. Pers. Individ. Dif. 126, 12–18. doi: 10.1016/j.paid.2018.01.009

[ref61] Yang X. X. (2019). Research on the influence of “information cocoon house” and internet culture on lifestyle. News Res. 20191, 19–22. doi: 10.3969/j.issn.1003-3629.2019.01.004

[ref62] Yue A. Gao J. Yang M. Swinnen L. Medina A. Rozelle S. (2018). Caregiver depression and early child development: a mixed-methods study from rural China. Front. Psychol. 9:2500. doi: 10.3389/fpsyg.2018.02500, PMID: 30618931 PMC6295552

[ref63] Zhang D. D. Lu M. H. Wang W. H. (2019). The psychological demand theory of community corrections—based on the grounded theory. Psychol. Sci. 42, 237–244. doi: 10.16719/j.cnki.1671-6981.20190135

[ref64] Zhang Y. C. Zhou N. Cao H. Liang Y. Yu S. Li J.. (2019). Career-specific parenting practices and career decision-making self-efficacy among Chinese adolescents: the interactive effects of parenting practices and the mediating role of autonomy. Front. Psychol. 10:363. doi: 10.3389/fpsyg.2019.00363, PMID: 30846959 PMC6393363

[ref65] Zou X. Y. Jia Y. M. (2008). The research on the characteristics of autonomy development in middle school students. Educ. Sci. 24, 50–54. doi: 10.3969/j.issn.1002-8064.2008.05.011

